# Suppression of NLRP3 inflammasome orchestrates the protective efficacy of tiron against isoprenaline-induced myocardial injury

**DOI:** 10.3389/fphar.2024.1379908

**Published:** 2024-08-15

**Authors:** Doaa Abdelrahaman, Ola A. Habotta, Ehab S. Taher, Eman S. El-Ashry, Iman Ibrahim, Ahmed Abdeen, Ateya M. Ibrahim, Reham M. Ibrahim, Hala Anwer, Ostan Mihaela, Rada Olga, Khairiah M. Alwutayed, Rasha H. Al-Serwi, Mohamed El-Sherbiny, Safwa M. Sorour, Dalia H. El-Kashef

**Affiliations:** ^1^ Department of Internal Medicine, College of Medicine, Princess Nourah bint Abdulrahman University, Riyadh, Saudi Arabia; ^2^ Department of Forensic Medicine and Toxicology, Faculty of Veterinary Medicine, Mansoura University, Mansoura, Egypt; ^3^ Department of Basic Medical and Dental Sciences, Faculty of Dentistry, Zarqa University, Zarqa, Jordan; ^4^ Department of Pharmacology, Faculty of Veterinary Medicine, Mansoura University, Mansoura, Egypt; ^5^ Department of Pathology, Faculty of Veterinary Medicine, Mansoura University, Mansoura, Egypt; ^6^ Department of Forensic Medicine and Toxicology, Faculty of Veterinary Medicine, Benha University, Toukh, Egypt; ^7^ Department of Administration and Nursing Education, College of Nursing, Prince Sattam bin Abdulaziz University, Al-Kharj, Saudi Arabia; ^8^ Department of Family and Community Health Nursing, Faculty of Nursing, Port-Said University, Port Said, Egypt; ^9^ Department of Physiology, Faculty of Medicine, Benha University, Benha, Egypt; ^10^ Department of Biology, Faculty of Agriculture, University of Life Sciences"King Michael I" from Timisoara, Timisoara, Romania; ^11^ Department of Biology, College of Science, Princess Nourah bint Abdulrahman University, Riyadh, Saudi Arabia; ^12^ Department of Basic Dental Sciences, College of Dentistry, Princess Nourah bint Abdulrahman University, Riyadh, Saudi Arabia; ^13^ Department of Basic Medical Sciences, College of Medicine, AlMaarefa University, Riyadh, Saudi Arabia; ^14^ Department of Pharmacology, Faculty of Medicine, Benha University, Benha, Egypt; ^15^ Department of Pharmacology and Toxicology, Faculty of Pharmacy, Mansoura University, Mansoura, Egypt

**Keywords:** inflammatory cytokines, isoproterenol, myocardial infarction, NOD-like receptor protein 3 inflammasome, oxidative stress, tiron

## Abstract

The major contribution of myocardial damage to global mortalities raises debate regarding the exploration of new therapeutic strategies for its treatment. Therefore, our study investigated the counteracting effect of tiron against isoprenaline (ISO)-mediated cardiac infarction in mice. Tiron was administered to mice for 7 days prior to two consecutive injections of ISO on days 8 and 9 of the treatment protocol. Tiron significantly reduced the levels of CK-MB, LDH, and AST in serum samples of ISO-challenged mice. A considerable increase in the cardiac antioxidant response was observed in tiron-treated mice, as indicated by depletion of MDA and enhancement of antioxidant activities. Furthermore, tiron induced a marked decrease in NLRP3, ASC, and caspase-1 levels accompanied by weak immune reactions of IL-1β, NF-κB, TLR4, and iNOS in the infarct cardiac tissues. Histopathological screening validated these variations observed in the cardiac specimens. Thus, tiron clearly mitigated the oxidative and inflammatory stress by repressing the NLRP3 inflammasome and the TLR4/NF-κB/iNOS signaling cascade.

## 1 Introduction

Isoproterenol (ISO) is a synthetic non-selective β-adrenergic receptor agonist and an isopropylamine analog of adrenaline (Song et al., 2020; Pandi et al., 2022). Since ISO controls myocardial contractility and metabolism, it is frequently used to treat heart block, cardiac arrest, bradycardia, and occasionally asthma (Timercan et al., 2019; Asiwe et al., 2023). However, cardiotoxicity is a common side effect of ISO (Pandi et al., 2022). Oxidative stress and the production of oxygen-derived free radicals are considered to be the primary causes of the many and varied processes underlying ISO-induced myocardial damage (Obeidat et al., 2022; Asiwe et al., 2023). Myocardial infarction (MI) is one of the leading causes of death in cardiac disorders (Meeran et al., 2020). Accumulating evidence suggests that oxidative and inflammatory stresses play substantial roles in the pathogenesis of MI (Cinar et al., 2021). Highly reactive radicals could evoke lipid peroxidation and trigger cell death *via* multiple pathways, such as apoptosis and autophagy (Zou et al., 2022). ISO has been implicated in these types of destructive mechanisms in damaged heart tissue (Hamed et al., 2020).

Notably, the NOD-like receptor protein 3 (NLRP3) inflammasome has been implicated in the inflammatory response in numerous diseases such as renal ischemic/reperfusion injury, diabetes, tumors, and atherosclerosis (Ding et al., 2019; Lei et al., 2022). Upon activation, the NLRP3 inflammasome activates caspase-1 and apoptosis-associated speck-like protein, ASC (Schulz et al., 1995; Yu et al., 2018). Subsequently, caspase-1 and ASC stimulate the generation of related inflammatory cytokines, including interleukin-1β (IL-1β), which are critically involved in ischemic injury (Shao et al., 2022). Previous reports have established that cardiac ischemic injury is mediated through stimulation of the NLRP3 inflammasome in various animal models (Xiao et al., 2020; Chen et al., 2022; Lei et al., 2022). Toll-like receptor 4 (TLR4) exacerbates ischemic tissue damage by triggering nuclear factor kappa B (NF-κB) signaling, which increases the expression of inflammation-related genes (Othman et al., 2022; Kassab et al., 2022; Wang et al., 2022; Habotta O. A. et al., 2023). Additionally, inhibition of NF-κB transcription has been reported to repress activation of the NLRP3 inflammasome and attenuate proinflammatory mediators (Zhao et al., 2020; Hua et al., 2022; Habotta O. et al., 2023). Therefore, targeting the NF-κB/NLRP3/caspase-1 signaling pathway is a promising therapeutic approach for managing MI-related cardiac damage.

Numerous therapeutic agents have been developed for heart diseases, but these agents are associated with undesirable adverse effects and expensive costs (Khan et al., 2022). Tiron is a non-toxic, water-soluble alpha-tocopherol analog. It is an effective chelating agent as it forms a water-soluble complex with metal ions (Mohamed et al., 2021). Previous reports have documented its effectiveness in mitigating various metal toxicities, including magnesium and titanium (Morgan et al., 2017; Abdel-Magied et al., 2019). Tiron possesses potent ROS scavenging action by removing ROS inside mitochondria where tiron is located (Abdel-Magied et al., 2019). Morgan et al. (2017) found that tiron protected rat kidneys from exposure to titanium oxide nanoparticles by increasing antioxidants and decreasing lipid peroxidation. Furthermore, tiron mitigated oxidant and inflammatory responses in the pulmonary tissue of asthmatic mice challenged with ovalbumin (El-Sherbeeny et al., 2016). In an acute pancreatitis model using rats, tiron had significant antioxidant effects in the pancreas, liver, lung, and kidney (Ateyya et al., 2016).

Based on these considerations, our study expanded our understanding of the ameliorative effects of tiron on cardiac injury induced by ISO. ISO induces a pathological condition that mimics heart injuries, including MI in humans. This study evaluated cardiac, oxidant, and inflammatory biomarkers, as well as histopathological changes that occurred in cardiac tissues.

## 2 Materials and methods

### 2.1 Chemicals and experimental animals

Male Swiss albino mice weighing between 15 and 20 g were used in the current experiment. All mice were purchased from the Animal Unit of Mansoura University, Egypt. They were kept in a conventional laboratory setting with a 12-h light and dark cycle, a temperature range of 23°C–25°C, and a relative humidity facility. They were given free access to water and a balanced diet. ISO was obtained from Sigma-Aldrich, MO, United States and tiron was purchased from Acros Organics, Geel, Belgium, and then it was dissolved in normal saline.

### 2.2 Experimental protocol and sampling

Four groups of mice (*n* = 5 mice each) were randomly assigned as follows: a CTL group, where mice received only normal saline and served as negative controls; an ISO group, where mice were injected with ISO hydrochloride (85 mg/kg body weight, subcutaneously); an ISO + T1 group, where mice received ISO (85 mg/kg body weight, subcutaneously) and tiron (140 mg/kg body weight, intraperitoneally); and an ISO + T2 group, where mice were given ISO (85 mg/kg body weight, subcutaneously) and tiron (280 mg/kg body weight, intraperitoneally) (Mohamed et al., 2021; Chu et al., 2021). Mice were administered tiron for 7 consecutive days, while ISO was given on days 8 and 9.

Mice were sedated with secobarbital (Sigma-Aldrich, MI, United States) at a dose rate of 50 mg/kg, i. p., and blood samples were collected from the radio-orbital plexus. The heart tissues were collected from all treated groups 24 h after the last ISO management. Subsequently, each mouse was decapitated, and the heart was dissected and divided into two parts. The first part was used to prepare a 10% (wt/vol) homogenate by directly mixing the tissue with ice-cold 10 mM phosphate buffer (pH 7.4) and then centrifuged at 4 C for 10 min. The supernatant was used for biochemical tests. The second part was preserved in buffered formalin for histopathological assessment.

### 2.3 Determination of cardiac enzymes

Analysis of aspartate transaminase (AST), creatine kinase–myoglobin binding (CK-MB), and lactate dehydrogenase (LDH) activities was determined using colorimetric kits that were acquired from Human (Wiesbaden, Germany).

### 2.4 Measurement of cardiac oxidant/antioxidant parameters

Malondialdehyde (MDA) content and the enzymatic activities of superoxide dismutase (SOD) and catalase (CAT) were estimated based on the manufacturer’s protocols.

### 2.5 Assessment of cardiac inflammatory biomarkers

The assessment of inflammation in the cardiac homogenates was achieved using ELISA kits for caspase-1 (MY-Bio-Source Co., United States), NLRP3 (Aviva Systems Biolog, United States), and ASC (LifeSpan Biosciences Co., United States), according to the manufacturer’s protocols.

### 2.6 Histopathological examination

The myocardial tissues were dissected from the mice and immediately fixed in 10% neutral buffered formalin for 24 h. The fixed tissue was processed and embedded in paraffin wax. Embedded samples were cut into 5 µm thickness. The sliced heart sections were stained using hematoxylin and eosin. The left ventricle focusing on the deep muscle layer was examined under a light microscope (Olympus CX 31 microscope, Tokyo, Japan). The necroinflammatory lesions were quantitatively scored in the different experimental groups using ImageJ software.

### 2.7 Immunohistochemistry

IL-1β, inducible nitric oxide synthase (iNOS), NF-κB, and TLR4 expression were assessed in the fixed mouse heart tissues using standard protocols (Buchwalow and Böcker, 2010). Here, 5 µm tissue sections of paraffin-embedded specimens were deparaffinized in an oven (70°C–75°C) for 20 min. The deparaffinized sections underwent antigen retrieval for 30 min in a hot water bath, followed by 10 min of cooling at room temperature. After 30-min incubation in hydrogen peroxide (0.3%), the sections were treated with a preblocking solution for 10 min and then washed with phosphate-buffered saline (PBS) to prevent non-specific antibody binding. The sections were treated with a 1:100 dilution of rabbit anti-caspase 3 polyclonal antibody for 1 hour in a humid environment. After washing, the sections were incubated in a biotinylated secondary antibody for 10 min with additional incubation with streptavidin labeled with horseradish peroxidase (HRP). The sections were washed with PBS and incubated for 5 minutes at ambient temperature in a dark, humid environment using a DAB substrate chromogen system. Subsequently, they were rinsed with tap water. After cleaning the sections with xylol, they were counterstained for 10–20 min with hematoxylin, rinsed in distilled water, rinsed in acid ammonia water, dehydrated in a graded alcohol series, and cover-slipped. The sections were examined, and images were captured using an Olympus CX31 microscope (Tokyo, Japan). The positive stained area was semi-quantitatively assessed in the different experimental groups using ImageJ software.

### 2.8 Molecular docking

The three-dimensional structures of mice’s β1AR, β2AR, β3AR, caspase-1, NLRP3, and ASC were retrieved from the UniProt database (https://www.uniprot.org/). The solvent was removed, and polar hydrogens were added by MOE docking software. Moreover, the three-dimensional structure of ISO was retrieved from the PubChem database (https://pubchem.ncbi.nlm.nih.gov/). Molecular docking interactions and visualization were performed using MOE software.

### 2.9 Statistical analyses

Means ± SE were used to represent the results. One-way ANOVA was used to carry out statistical comparisons of the various groups. Duncan’s test was used as a *post hoc* test to compare between groups, with a *p*-value less than 0.05 set as the limit for significance. RStudio were used to analyze and visualize the data.

## 3 Results

### 3.1 Impact of tiron on the cardiac enzyme activities in ISO-induced mice

**FIGURE 1:**
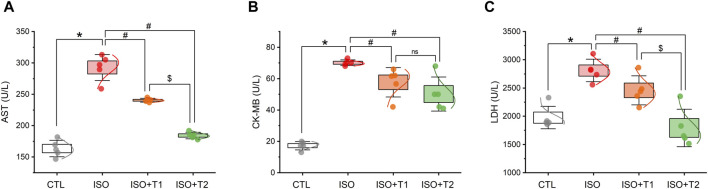
Effect of tiron on ISO-induced myocardial injury markers. **(A)** Serum AST, **(B)** serum CK-MB, and **(C)** serum LDH. AST, aspartate transaminase enzyme; CK-MB, creatine kinase-MB; LDH, lactate dehydrogenase enzyme. Data are presented as means ± SE (*n* = 5). *p < 0.05 vs. CTL group; ^*#*^ *p* < 0.05 vs. ISO group; and ^*$*^ *p* < 0.05 vs. ISO + T1.

We examined the activity of myocardial injury markers in serum samples to determine whether tiron could lessen ISO-induced myocardial injury. As depicted in Figure 1, compared to healthy controls, ISO injection induced cardiac dysfunction, as indicated by significant increases in CK-MB, LDH, and AST activity; tiron administration reduced their activity in this animal model. The LDH activity in the ISO + T1 and ISO groups showed no appreciable difference. In addition, no discernible difference in the CK-MB levels was observed when comparing the ISO + T1 and ISO + T2 groups. Noticeably, the tiron administration decreased the AST activity in a dose-dependent manner.

### 3.2 Effect of tiron on cardiac oxidative status and antioxidant biomarkers

**FIGURE 2:**
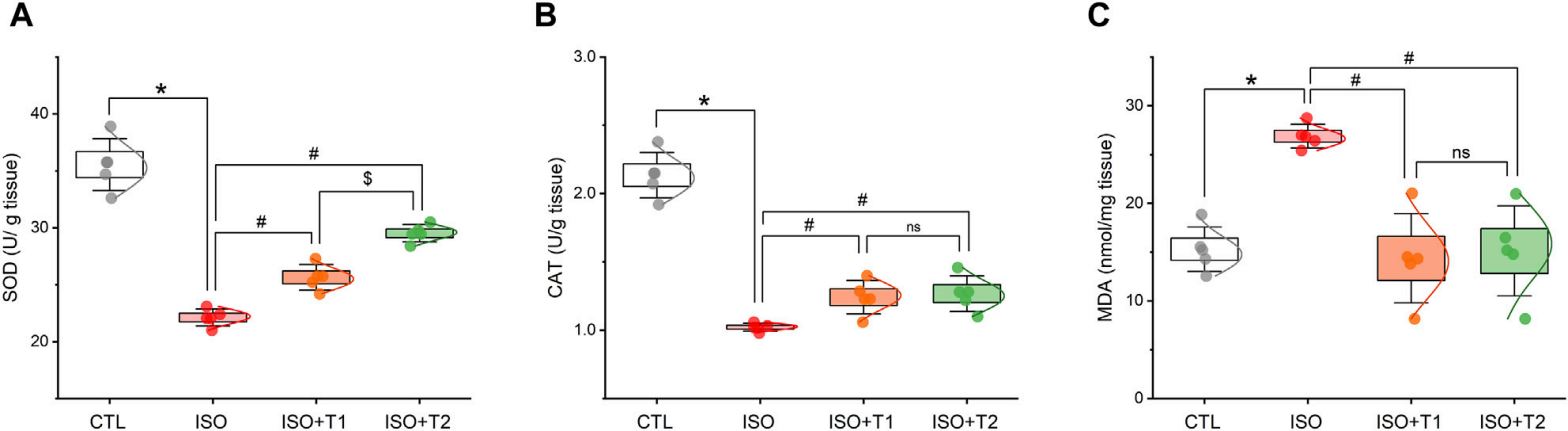
Effects of tiron on ISO-induced myocardial oxidant/antioxidant markers. **(A)** SOD activity, **(B)** CAT activity, and **(C)** MDA concentration. SOD, superoxide dismutase; CAT, catalase; MDA, malondialdehyde. Data are presented as means ± SE (*n* = 5). *p < 0.05 vs. CTL group; ^*#*^ *p* < 0.05 vs. ISO group; and ^*$*^ *p* < 0.05 vs. ISO + T1.

As seen in Figure 2, a considerable increase in MDA, a marker of lipid peroxidation, was observed in hearts from the ISO group compared to the control mice. Furthermore, the mice that received ISO exhibited lower antioxidant activity levels of SOD and CAT. Tiron, however, markedly decreased the cardiac MDA content and elevated SOD and CAT activities compared to ISO-treated mice. On the other hand, no discernible differences were observed in MDA and CAT between the ISO + T1 and ISO + T2 groups. These findings indicate that ISO treatment caused oxidative stress in the hearts of mice, which was suppressed by tiron treatment.

### 3.3 Effect of tiron on NLRP3 inflammasome activation

**FIGURE 3:**
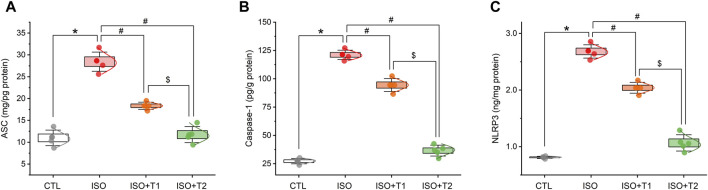
Effects of tiron on ISO-induced myocardial inflammatory markers. **(A)** ASC level, **(B)** caspase-1 level, and **(C)** NLRP3 level. ASC, apoptosis-associated speck-like protein containing a CARD; NLRP3, NOD-like receptor protein 3. Data are presented as means ± SE (*n* = 5). *p < 0.05 vs. CTL group; ^*#*^ *p* < 0.05 vs. ISO group; and ^*$*^ *p* < 0.05 vs. ISO + T1.

The protein levels of NLRP3, ASC, and caspase-1 were assessed in the heart specimens to clarify the effect of tiron on the NLRP3 inflammasome in MI. The activation of NLRP3 caused by the injection of ISO was accompanied by marked increases in cardiac caspase-1, NLRP3, and ASC expression levels. Furthermore, tiron treatment markedly decreased the upregulated proteins compared to the ISO group (Figure 3).

### 3.4 Multivariate analyses

**FIGURE 4:**
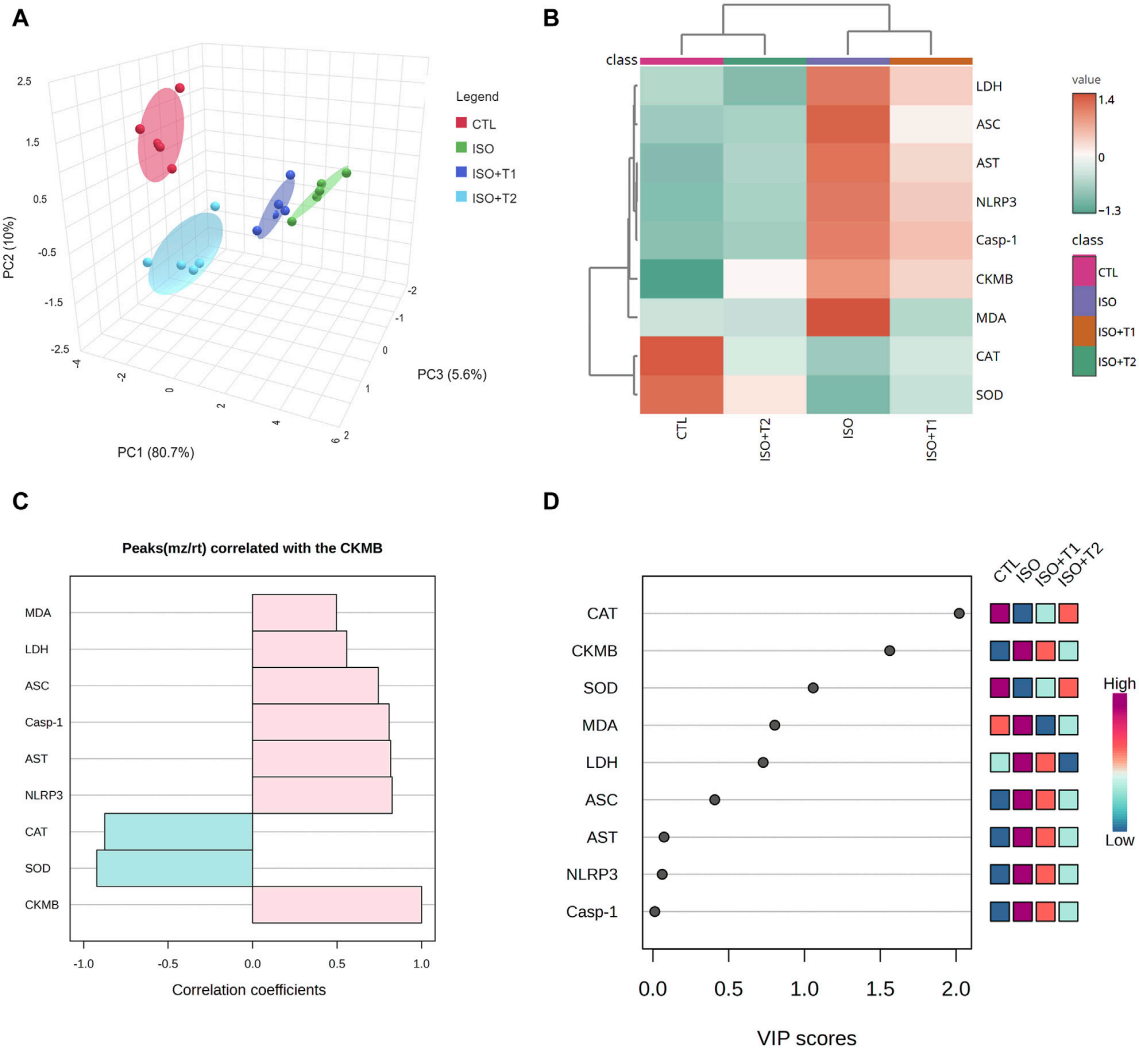
Multivariate analyses of all data sets after ISO and tiron intervention. **(A)** Three-dimensional scoring plot of principal component analysis (PCA). **(B)** Clustering heatmap for the variable averages and various groups; the concentration levels are illustrated by each colored cell on the map. **(C)** Hunter heatmap. **(D)** Variable important project scores.

As depicted in Figure 4A, principal component analysis (PCA) was used to analyze the overall data and evaluate the relationship between the different interventions and covariates. The 3D PCA revealed three dimensions for all variables, which accounted for 96.3% of the variation. Although dimensions 2 (10%) and 3 (5.6%) revealed the lowest variance proportions, dimension 1 provided most of the variation (80.7%). PCA indicated that the ISO-injured mice were distinctively separated from the other treated mice, including CTL, ISO + T1, and ISO + T2 on the opposite side. Therefore, mice pretreated with tiron exhibited a remarkable difference compared to mice given ISO.

The clustering heatmap (Figure 4B) illustrates an intuitive visualization of the entire data set, highlighting and summarizing the concentration values of all assessed markers following different interventions. ISO-injured heart tissue was more likely to show damage, according to the given parameters than in the other groups (CTL, ISO + T1, and ISO + T2). The tiron and ISO co-administered groups displayed intermediate color intensities for all measured parameters, indicating that animals treated with ISO responded positively to the protective action of the tiron pretreatment in a dose-dependent manner. Since CK-MB is a commonly approved marker for cardiac injury, a hunter heatmap (Figure 4C) was generated to explore the correlation between the degree of heart injury and other concentration values of measured variables. These data indicated that NLRP3 and SOD exhibited the strongest positive and negative correlations, respectively, with the degree of damage. In addition, as seen in Figure 4D, the variable importance in projection (VIP) scores indicated that CAT, CK-MB, SOD, MDA, LDH, ASC, AST, and NLRP3 had a considerable effect in the existing study, with scores up to 2.0.

### 3.5 Histopathological findings

**FIGURE 5:**
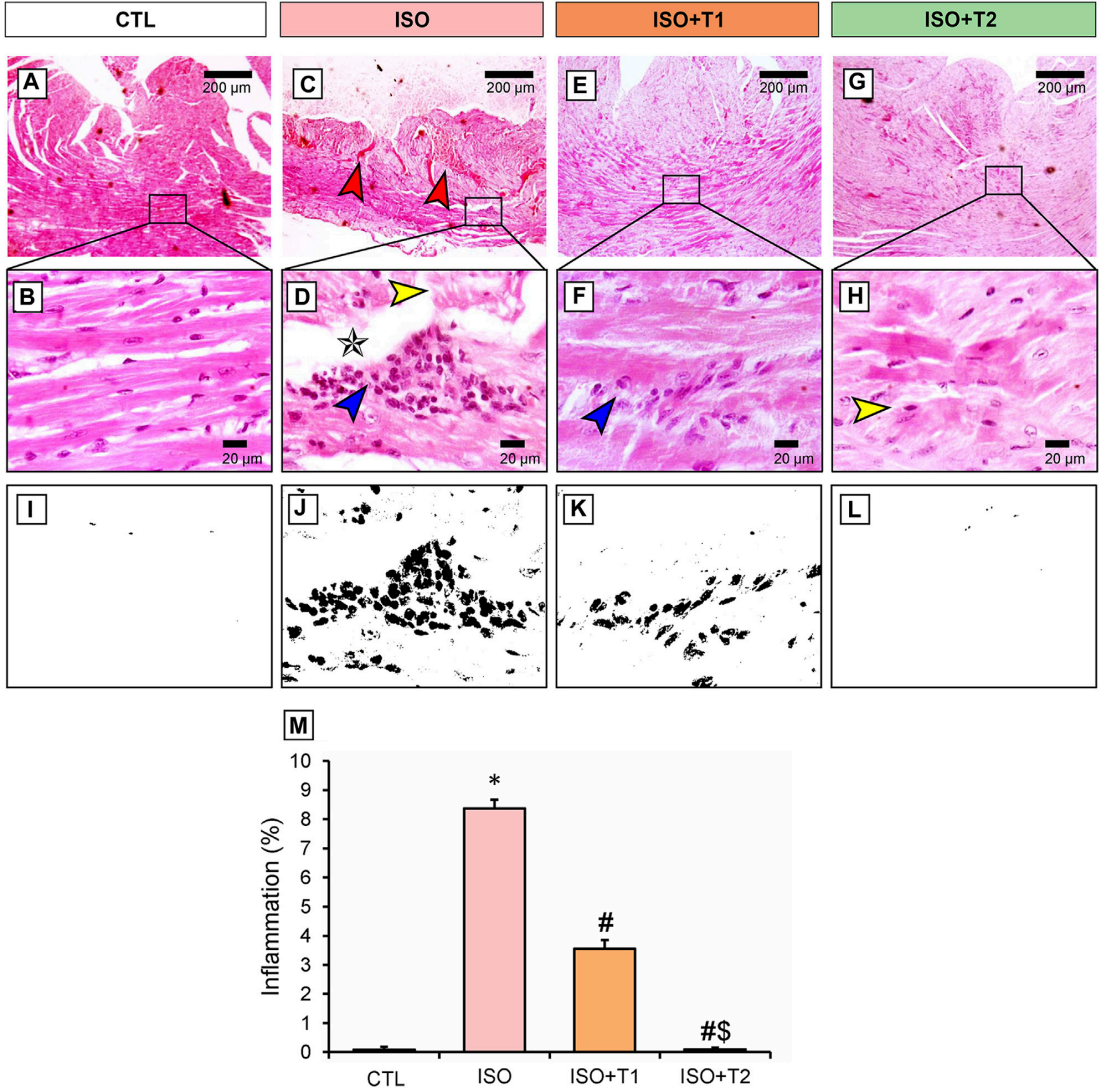
Representative photomicrograph of the heart section from different treatment groups. **(A)** Control cardiac muscle presents normal histological appearance of muscle fibers with centrally located nucleus. **(B)** Higher magnification of A (Scale bar = 20 μm). **(C, D)** ISO group shows extensive vascular congestion (red arrow) with edema (star) separated a necrotic muscle fiber (yellow arrow) with loss of striation and multifocal-to-coalescing inflammatory aggregates (blue arrow). **(D)** Higher magnification of C (Scale bar = 20 μm). **(E, F)** ISO + T1 exhibits few inflammatory aggregates replaced an occasional necrotic myocyte (blue arrow). **(E)** Higher magnification of F (Scale bar = 20 μm). **(G, H)** ISO + T2 indicates partial restoration of cardiac architecture with few necrotic myocytes (yellow arrow) and without any inflammation. **(H)** Higher magnification of G (Scale bar = 20 μm). **(I–M)** Corresponding qualitative image and semi-quantitative analysis of inflammation. Data are presented as means ± SE. *p < 0.05 vs. CTL group; ^*#*^ *p* < 0.05 vs. ISO group; and ^*$*^ *p* < 0.05 vs. ISO + T1.

The H&E-stained heart section exhibited a normal myocardial architecture in the control group (Figures 5A, B, I). However, high necroinflammation was observed in the ISO group compared to other groups. In addition, marked extensive vascular congestion with edema-separated necrotic muscle fibers, loss of striation, multifocal-to-coalescing inflammatory aggregates were detected in the ISO group (Figures 5C, D, J). On the other hand, mice treated with ISO and low doses of tiron (ISO + T1) presented a remarkable reduction of ISO deleterious effect with moderate myonecrosis and inflammation (Figures 5E, F, K). Interestingly, same findings but with better improvements were documented in the ISO + T2 group, which was attested by the scoring data indicated in Figure 5M.

### 3.6 Assessment of inflammation

**FIGURE 6:**
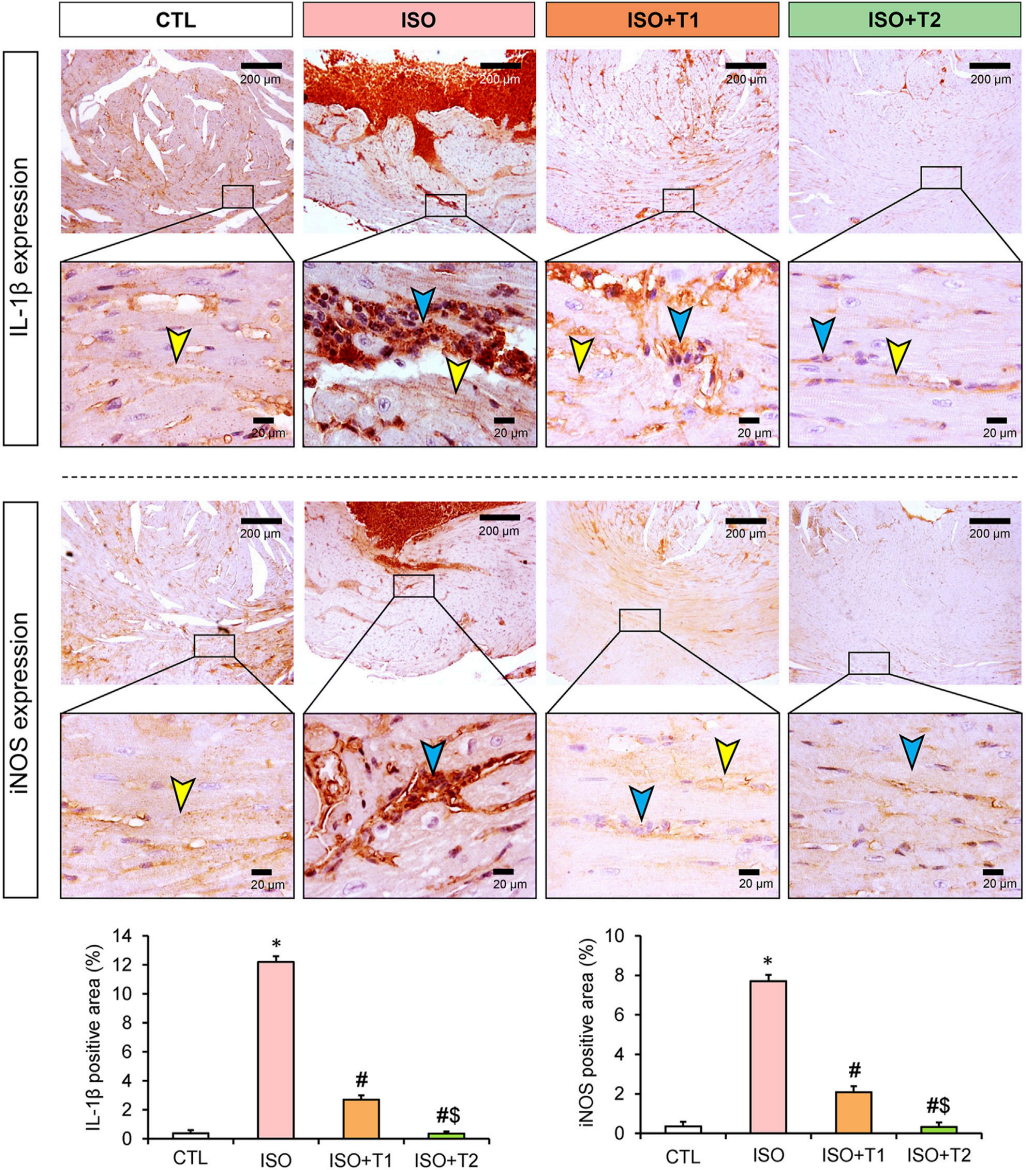
Representative immunohistochemical staining of IL-1β and iNOS expressions in heart tissue at low and high magnification scales (scale bars = 200 and 20 μm, respectively). The blue arrow indicates the positive immunostained inflammatory cells, and the yellow arrow points the positive immunostained myocytes. Bar plots show the semi-quantitative expression levels of IL-1β and iNOS presented in the upper images. Data are presented as means ± SE. *p < 0.05 vs. CTL group; ^*#*^ *p* < 0.05 vs. ISO group; and ^*$*^ *p* < 0.05 vs. ISO + T1.

**FIGURE 7:**
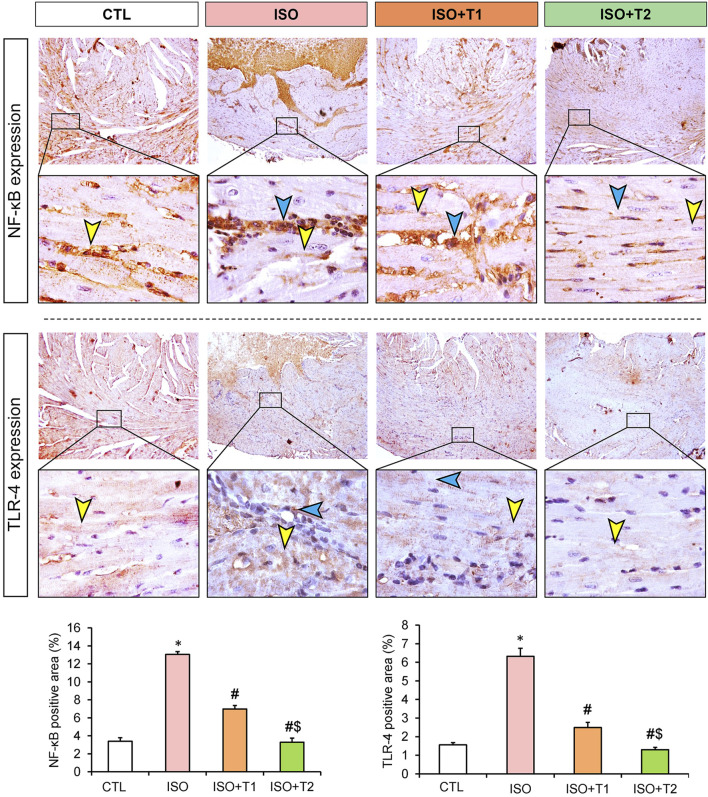
Representative immunohistochemical staining of NF-κB and TLR4 expressions in heart tissue at low and high magnification scales (scale bars = 200 and 20 μm, respectively). The blue arrow indicates the positive immunostained inflammatory cells, and the yellow arrow points the positive immunostained myocytes. Bar plots show the semi-quantitative expression levels of NF-κB and TLR4 presented in the upper images. Data are presented as means ± SE. *p < 0.05 vs. CTL group; ^*#*^ *p* < 0.05 vs. ISO group; and ^*$*^ *p* < 0.05 vs. ISO + T1.

The inflammatory markers, IL-1β, iNOS, NF-κB, and TLR4 exhibited significant reductions in the ISO + T1 and ISO + T2 groups compared to the ISO group (Figures 6, 7). In contrary, the control animals showed weak cytoplasmic IL-1β, iNOS, and TLR4 expressions in the sarcoplasm of cardiac muscle fibers with cytoplasmic and nuclear expression of NF-κB. However, strong expression of these inflammatory markers in the inflammatory aggregates that replaced the necrotic myocytes with faint expression in the surrounding myocytes was recorded. The semi-quantitative data obtained from immunostaining of those proteins indicated significant differences between the ISO group and other treated groups, and a dose-dependent response was seen between the ISO + T1 and ISO + T2 groups (Figures 6, 7).

### 3.7 Molecular docking

**FIGURE 8:**
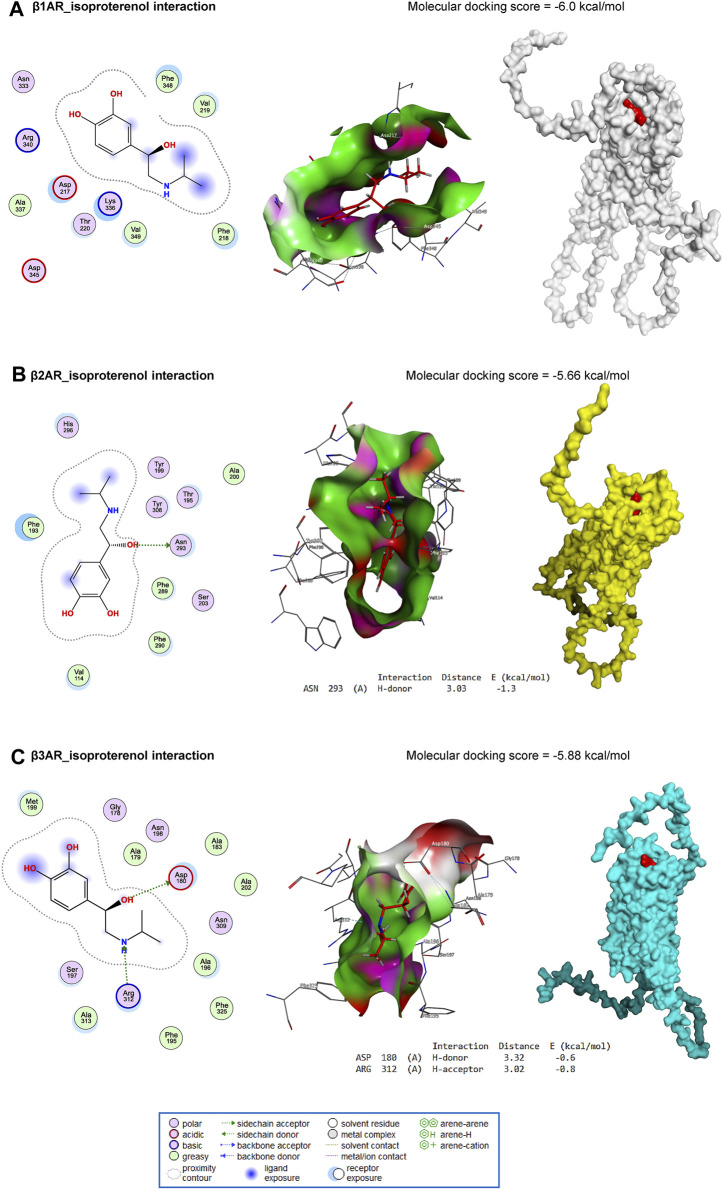
Molecular docking interaction of ISO against mouse β1AR **(A)**, β2AR **(B)**, and β3AR **(C)**.

ISO interacted with the binding site of β1AR with an energy of −6.0 kcal/mol (Figure 8A). In addition, ISO bound with ASN293 (H-donor) residue in the binding site of β2AR with an energy of −5.66 kcal/mol (Figure 8B). Similarly, with an energy of −5.88 kcal/mol, ISO interacted with ASP180 (H-donor) and ARG312 (H-acceptor) residues in the binding site of β3AR (Figure 8C).

**FIGURE 9:**
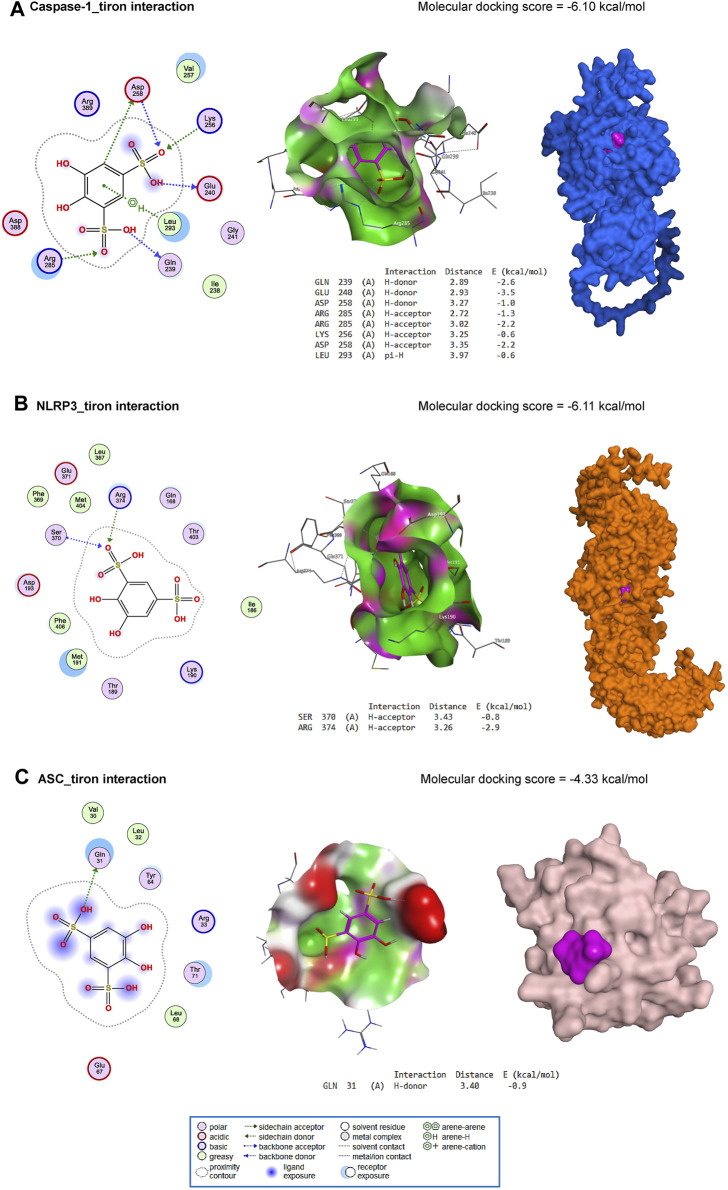
Molecular docking interaction of tiron against mouse caspase-1 **(A)**, NLRP3 **(B)**, and ASC **(C)**.

Furthermore, tiron interacted with the binding site of caspase-1 by H-donor (GLN239, GLU240, and ASP258 residues), H-acceptor (ARG285, LYS256, and ASP258 residues), and pi-H (LEU293 residue) with a binding energy of −6.10 kcal/mol (Figure 9A). Furthermore, tiron bound with SER370 (H-acceptor) and ARG374 (H-acceptor) residues in the binding site of NLRP3 with an energy of −6.11 kcal/mol (Figure 9B). With −4.33 kcal/mol of energy, tiron interacted with the GLN31 (H-donor) in the binding site of ASC, as represented in Figure 9C.

## 4 Discussion

Myocardial damage is recognized as a leading cause of morbidity and mortality worldwide (Ardjmand et al., 2019). Even though several medical and interventional treatments have recently seen substantial advancements, many patients still have poor prognoses, lowering their quality of life and increasing the risk mortality (Hamed et al., 2020). Isoprenaline (ISO) is a catecholamine that stimulates β1 adrenergic receptors, disrupting myocardial contractions under stressful situations that can lead to heart failure (Shahzad et al., 2019). ISO was reported to induce cardiac abnormalities in experimental animals similar to the pathogenic pathways observed in humans. Thus, it offers an appropriate experimental model for investigating the beneficial effects of various medications (Hamed et al., 2020). In this study, the possible protective effects of tiron against ISO-mediated myocardial injury were investigated by focusing on the NLRP3 inflammasome and TLR4/NF-κB/iNOS signaling. We observed that ISO exposure caused severe myocardial damage that was verified by alterations in heart function and structure. Several studies have suggested that ISO produces myocardial necrosis through inducing ischemia (Malik et al., 2011; Ojha et al., 2013). This concurs with our study, where marked myonecrosis, infiltration of inflammatory cells, edema, and hemorrhage were observed. However, mice treated with tiron (140 mg/kg) exhibited a marked reduction in myocardial damage, as demonstrated by the presence of minimal, occasional necrosis, inflammation, and decreased edema. Mice treated with tiron (280 mg/kg) also showed minimal-to-mild myocardial necrosis, inflammation, and edema.

Regarding heart function, ISO injection substantially increased the enzymatic activities of heart injury markers (CK-MB, AST, and LDH). It has been reported that the myocardial cells contain LDH and CK under normal conditions (Song et al., 2020). However, when these cells are destroyed under pathological conditions such as disturbed metabolism and inadequate oxygen delivery, these enzymes leak into the bloodstream (Song et al., 2020; Al-Brakati et al., 2021). Administration of tiron at both doses markedly decreased the serum activity of CK-MB compared to the ISO group. Concerning LDH activity, only tiron at the highest dose succeeded in restoring normal LDH activity. These observations suggest that tiron has a membrane-stabilizing effect in the myocardium of treated mice.

It is well-known that ISO produces deleterious oxidative insults in myocardial tissue, which evokes infarct-like damage in the heart. Numerous processes have been proposed to explain how ISO might cause myocardial injury, and one of the most important causes is the formation of ROS during catecholamine autoxidation (Mert et al., 2018). Similarly, our results revealed that ISO caused disturbance in the oxidant/antioxidant balance, as demonstrated by a significant elevation in MDA content and reduction in the activities of SOD and CAT compared to the controls. Notably, pretreatment with tiron significantly decreased the MDA content and increased the activity of both enzymes compared to ISO-treated mice. This indicates that tiron might protect the heart against lipid peroxidation by eliminating surplus damaging free radicals induced by ISO.

Earlier studies have clarified the crucial function that oxidative stress plays in the NLRP3 inflammasome (Abais et al., 2015; El-Kashef and Serrya, 2019). Thus, suppression of oxidative stress could prevent NLPR3 inflammasome activation. In this work, ISO induced significant increases in cardiac levels of NLRP3, ASC, caspase-1, and protein expression of IL-1β compared to the controls. However, pretreatment with tiron resulted in a profound reduction in their protein expression levels compared to the ISO group. These findings suggest that tiron might exert anti-inflammatory effects by blocking the NLRP3 pathway.

The inflammatory response during myocardial infarction is regulated by several inflammatory markers, including IL-1β, iNOS, NF-κB, and TLR4 (Viswanadha et al., 2020). This investigation detected notable increases in the immune reactions of IL-1β, iNOS, NF-κB, and TLR4 in cardiac tissue, following the ISO insult compared to the controls. On the other hand, tiron-treated groups showed mild-to-moderate expression of these inflammatory biomarkers when compared to the ISO group. Accordingly, the myocardial damage in the ISO-treated group could be attributed to the responsive mechanism of TLR4 to damage-associated molecular patterns (DAMPs), which promote upregulation of proinflammatory cytokines such as IL-1β (Liu et al., 2015). The elevation of proinflammatory regulators might hasten the progression of heart damage. Elevated IL-1β expression might enhance specific intracellular pathways, including NF-κB and ROS production in cardiomyocytes (Machida et al., 2003; Ha et al., 2011; Lokman et al., 2022). Furthermore, IL-1β activates iNOS in cardiac critical for myocardial contractility (Schulz et al., 1995).

The results obtained from the molecular docking experiment revealed comparable free energies of binding of ISO with β1AR, β2AR, and β3AR in mice, suggesting the potential impact of ISO on the myocardial function attested in the present study. The *in silico* study also revealed how tiron could directly interact with caspase-1, NLRP3, and ASC proteins. This interaction pattern was in harmony with the data obtained from the current *in vivo* experiment.

**FIGURE 10:**
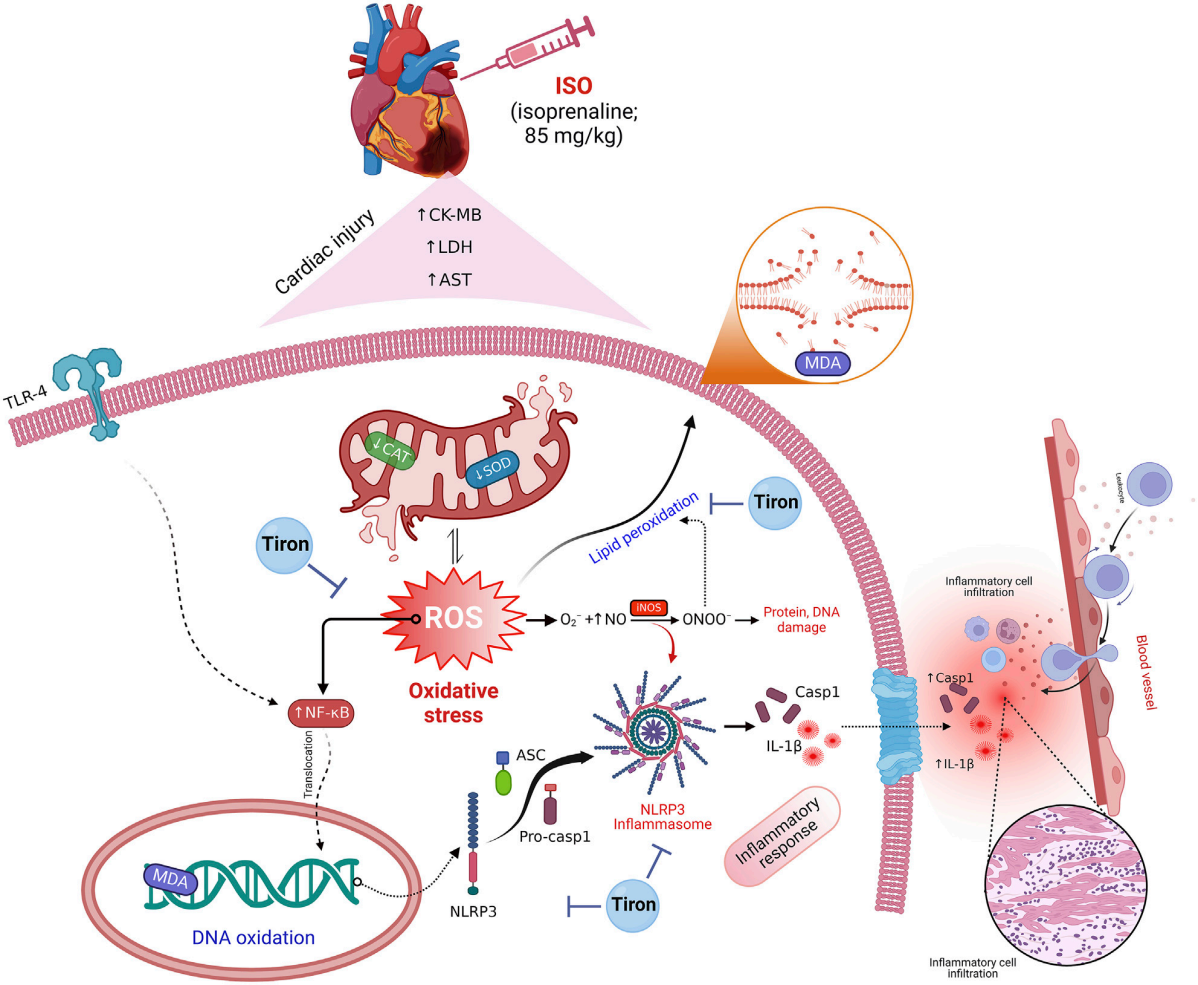
Proposed mechanistic insights involved in the protective activity of tiron against ISO cardiotoxicity.

The multivariate analyses provide an intuitive visualization of the entire data set, which summarize all measurements in one output and highlight the influence of all variables in the current study after different interventions. Herein, PCA, a clustering heatmap, a hunter heatmap, and VIP scores were created to assess the effects of the different interventions on cardiac tissue. The data obtained from PCA revealed that the toxic group (ISO) could influentially discriminate other treatments, where the ISO-injured mice were distinctly set apart from other mice by clustering independently on the far right along the PC1 axis. In the same data frame, the clustering heatmap illustrated the changes in the investigated variables due to ISO exposure along all studied parameters in all groups. It also emphasized the extreme alterations in the ISO-treated mice in comparison to the control mice. However, the ISO + T1 and ISO + T2 groups were located in the middle range among those of ISO and CTL groups with more pronounced effects in the ISO + T2-pretreated animals. Moreover, the strong positive or negative correlations between CK-MB and other oxidant/antioxidants, inflammation, and apoptotic markers exhibited by the hunter heatmap confirm the potential involvement of those selected measurements in the proposed mechanisms underlay the ISO-induced injury and the mitigating effects of tiron in the heart tissue. Alongside the VIP scores, those parameters are sorted in order according to their impact in this study; therefore, CAT, CK-MB, SOD, MDA, and LDH were the top influencing ones. Collectively, these data robustly corroborated the cardioprotective potential of tiron for ISO-induced heart injury. Figure 10 summarizes the proposed mechanistic insights involved in the protective activity of tiron against ISO-induced cardiotoxicity.

## 5 Conclusion

Collectively, the myocardial preservation in the tiron-treated groups could be attributed to increased antioxidant capacity inflammation inhibition. These actions suggest that the enhancement of the cardiac antioxidant defense was mediated by increased levels of SOD and CAT concurrent with decreased MDA levels. Moreover, suppression of TLR4/NF-κB/iNOS and the NLRP3/caspase-1/IL-1β signaling pathways mediated the cardioprotective effect of tiron. Therefore, tiron could potentially be a valid, safe, and therapeutic substance for myocardial infarction. Further clinical studies are necessary to verify its clinical use in cases of myocardial infarction.

## Data Availability

The original contributions presented in the study are included in the article/Supplementary Material; further inquiries can be directed to the corresponding authors.
